# Governmental support for achieving “VISION 2020: the Right to Sight” in Iran: the cataract surgical rates

**DOI:** 10.1186/s12886-022-02559-9

**Published:** 2022-08-03

**Authors:** Hassan Hashemi, Farhad Rezvan, Abbasali Yekta, Mehdi Khabazkhoob

**Affiliations:** 1 Noor Research Center for Ophthalmic Epidemiology, Noor Eye Hospital, Tehran, Iran; 2 Noor Ophthalmology Research Center, Noor Eye Hospital, Tehran, Iran; 3 Department of Optometry, School of Paramedical Sciences, Mashhad University of Medical Sciences, Mashhad, Iran; 4 Department of Basic Sciences, School of Nursing and Midwifery, Shahid Beheshti University of Medical Sciences, Tehran, Iran

**Keywords:** Cataract surgery rate, Economic, Government, Cost

## Abstract

**Purpose:**

The aim of this study was evaluate the effect of governmental support in the form of Health Transformation Plan (HTP) on increasing the cataract surgical rate.

**Methods:**

The number of cataract surgeries was collected from Iranian cataract surgery clinics during 2019. HTP was implemented in 2014. Forty-seven major and forty-five minor surgery centers were selected from all provinces. In each center, sampling was done from 2012, 2013, 2015, and 2016.

**Results:**

On average, 6202 and 7134 cataract surgery rate were performed before and after HTP, respectively. The cataract surgical rate rose by 15.03% after the HTP. After HTP, the proportion of cataract surgery increased by 21.32% in governmental centers and decreased by 17.56%, 24.45%, and 14.89% in private, insurance, and charity centers, respectively. The cataract surgical rate was 4093 and 6026 in the first economic quartile (the poorest), 3669 and 4595 in the second quartile, 5884 and 5928 in the third quartile, and 8427 and 9681 in the fourth quartile (the richest) before and after HTP, respectively. The highest growth in the cataract surgical rate was seen in the first quartile (47.24%) followed by the second (25.26%), fourth (14.88%), and third quartiles (0.74%).

**Conclusion:**

The Health Transformation Plan has been successful in increasing the cataract surgical rate in the low-income group and identifying differences in the services as well as the economic groups within the population.

## Introduction

Recent reports indicate that 295 million people suffer from moderate to severe vision impairment and 43.3 are blind across the world [1]. The prevalence of vision impairment increased by 2.5% during 1990–2020 [1]. Moreover, it is estimated that 474 million patients will have moderate to severe vision impairment (38% increase) and 61 million people will be blind by 2050 [1].

Cataract is the leading cause of blindness and the second cause of vision impairment in the world [2]. A report indicated that 45% of blindness and 38.9% of vision impairment was attributable to cataract [2]. This is while surgery can easily restore vision and prevent blindness [3–5]. On the other hand, the elderly population is growing due to control of communicable diseases and increased life span [1]. Previous studies found a high prevalence of cataract in the elderly [6–10]. According to some studies, one in every two elderly people has cataract, the prevalence of cataract is reported to be as high as 87% in the age group over 60 years [6].

The WHO and the International Agency for the Prevention of Blindness launched a joint initiative entitled “VISION 2020: The Right to Sight” in 1999 to eliminate preventable blindness [11]. The WHO implemented a global action plan (GAP) for universal eye health in 2013 with the aim of reducing the prevalence of avoidable blindness and vision impairment by 25% by 2019 from the baseline established by the WHO in 2010 [12].

The cataract surgical rate (CSR) is one of the most important indicators for monitoring the GAP progress [12]. For this reason, CSR has been measured and evaluated in several studies and is considered a proxy for access to cataract services in different countries [12–19]. Previous studies suggest reported different CSRs that ranged between less than 500 and more than 10,000 surgeries per one million population [12]. CSR is higher in developed versus developing countries and has a direct correlation with the economic state, such that it is higher than 10,000 surgeries per one million population in developed countries and less than 500 surgeries per one million in countries like Kenya and Ethiopia [12].

Wong et al. [12] found a strong direct correlation between GDP and CSR in different countries. The CSR was as high as 10,500 surgeries per one million population in countries like France, the Netherlands, USA, and Sweden, with a GDP of at least 35,000 USD, while it was less than 2000 surgeries per one million in countries with a GDP of less than 8000 USD [12]. Considering the strong correlation between CSR and GDP and since the rich are tend to receive healthcare services in private centers, it seems that government support for offering surgical services and covering part of the expenses, especially in developing countries, can be associated with an increase in the CSR, resulting in a reduction in blindness and impaired vision [12].

In 2014, the Iranian Ministry of Health (MOH) implemented a Fundamental reform in the health sector known as the Health Transformation Plan (HTP) to improve the health system in governmental medical centers, provide financial support for people, improve equity in access to health services, and promote the quality of healthcare services [20].

One of the surgical procedures covered by the HTP is cataract surgery. The CSR has been already reported from Iran [21, 22]. However, due to the importance of the above, a study was conducted to evaluate the effect of implementing HTP on CSR in Iran and the trend of cataract surgery before and after HTP.

## Materials and methods

A cross-sectional routine database study was conducted in 2020.

### Sampling method

The methodology of this study was based on two reports already published from Iran [21, 22]. Briefly, using the MOH data, centers that offered cataract surgery during 2012–2016 were identified. All cataract surgery centers, including governmental, private, insurance, and charity centers were considered as the target population. According to the MOH data, 276 centers offer cataract surgery services in Iran. Centers that performed less than 100 cataract surgeries per year were removed from the sampling frame. Finally, 241 were selected as the sampling frame, of which 59 were major centers (more than 3000 cataract surgeries per year) and 182 were minor centers (less than 3000 surgeries per year). All major centers were selected, of which 54 participated in the study. Moreover, 47 minor centers were randomly selected proportional to the number of minor centers in each province, of which 45 centers agreed to participate. In total, 99 centers were studied.

### Training of data collectors

Trained staff collected the data from each center in coordination with the center’s medical records department. To evaluate the data validity and reliability, data were collected from four major and four minor centers twice, and the ICC between the number of surgeries from two reports was 0.979.

### Data collection

The first two weeks of the year are the New Year holidays in Iran when almost all outpatient clinics are inactive. For this reason, sampling was done from the whole year except for the first two weeks of each year. The sampling frame was 50 weeks per year. In each year, two weeks from each season were randomly selected for each center and the number of cataract surgeries during the two weeks was recorded (a total of eight weeks for each year).

### Health Transformation Plan (HTP)

The HTP was implemented in urban and rural centers in May 2014. The HTP was active in the following fields: 1- insuring the population lacking health insurance, 2- providing financial protection for patients admitted to hospitals affiliated with the Ministry of Health, 3- supporting the retention of physicians in underserved areas, and 4- completing and developing the family physician program and referral system. The HTP provided financial protection for patients at the time of receiving healthcare services from governmental hospitals affiliated with the Ministry of Health in the form of insuring patients lacking health insurance, which reduced the total hospitalization expenditures of patients to 6% in large cities and 3% in cities with less than 20 thousand population [20, 23–25]. Moreover, to expand healthcare services in different cities, measures such as equitable increase in healthcare services, support for retention of physicians in underserved areas, and family physician program and referral system were designed and implemented, following which improved the access of people from different economic classes living in different parts of the country to healthcare services, including eye care services.

To assess the effect of the HTP on the CSR, the two of two years before (2012 and 2013) and two years after implementing the HTP (2015 and 2016) were compared. Finally, the number of cataract surgeries performed in the four years (32 weeks) was collected from each center.

### Economic state

Provincial income was extracted from the data of the Statistical Center of Iran. The provinces were sorted from the lowest income to the highest income and categorized into four economic quartiles. The first quartile was the provinces with the lowest income.

In each center, 10 patient records with complete data were randomly selected from each year for visual acuity assessment. In other words, 40 medical records were selected from each center, totaling 3960 records. Visual acuity was recorded in LogMAR with a higher LogMAR value indicating a worse visual acuity.

### Data analysis

To calculate the CSR, the number of cataract surgeries performed in each center was determined. For this purpose, the number of surgeries in eight weeks was multiplied by 6.25 (since 8 out of 50 weeks were randomly selected). Then, because the number of selected minor and major centers was not proportional to the number of all centers across the country, a weight of 1.09 and 4.04 was considered for major (54/59) and minor centers (45/182) respectively, and the weight was multiplied by the number of surgeries performed in each center in each year. Finally, the cataract surgical rate per one million population per each year was calculated according to the country’s population in that year using the following formula. To evaluate the CSR according to the economic quartiles, the provinces were categorized into four quartiles, and the CSR was calculated according to the population in each quartile. To compare the CSR before and after HTP implementation, the average rate of 2013 and 2013 was compared with that of 2015 and 2016. To evaluate the trend of CSR in governmental, private, insurance, and charity centers, since the denominator for these centers was not clear, the proportion of cataract surgery before and after HTP was compared in each center.

$$\mathrm{CSR}=\frac{\begin{array}{c} \left[\mathrm{Number of cataract surgeries at major centers }\times 6.25 \times \mathrm{ weight of major centers}\right]\\ +[Number of cataract surgeries at minor centers \times 6.25 \times weight of minor centers]\end{array}}{\mathrm{Population of Iran in the given year}}$$

### Ethical issues

The Non communicable diseases unit of Ministry of Health, Treatment and Medical Education Tehran, Iran, approved the study protocol, which was conducted in accord with the tenets of the Helsinki Declaration.

## Results

Table 1 presents the number of cataract surgeries according to the study years and before and after HTP. The CSR was 6074, 6330, 6788, and 7479 cases per one million population in 2012, 2013, 2015, and 2016, respectively. The mean CSR was 6202 and 7134 per one million populations before and after HTP, respectively. According to the results, the number of cataract surgeries increased by 19.38% and the CSR improved by 15.03% after HTP.

**Table 1:** Number of cataract surgeries and cataract surgical rate (CSR) in Iran before and after Health Transformation Plan (HTP) in totally and according to economic quartiles

		Before HTP		After HTP			
		2012	2013	2015	2016	Before HTP^a^	After HTP^b^
Total	Number of cataract surgeries	462,102	487,496	535,856	597,802	474,799	566,829
	Population (1000)	76,075	77,016	78,940	79,926	76,546	79,433
	CSR per one million	6074	6330	6788	7479	6202	7134
	95% Confidence Interval / high	6063	6319	6778	7470	6192	7126
	95% Confidence Interval / low	6085	6341	6798	7489	6214	7146
Economic quartiles							
First	Number of cataract surgeries	39,713	42,084	57,687	64,457	40,899	61,072
	Population (1000)	9970	10,015	10,109	10,158	9993	10,134
	CSR per one million	3983	4202	5706	6345	4093	6026
Second	Number of cataract surgeries	50,925	56,311	63,442	75,472	53,618	69,457
	Population (1000)	14,530	14,694	15,024	15,193	14,612	15,109
	CSR per one million	3505	3832	4223	4968	3669	4595
Third	Number of cataract surgeries	131,267	134,777	134,543	144,927	133,022	139,735
	Population (1000)	22,448	22,762	23,404	23,732	22,605	23,568
	CSR per one million	5848	5921	5749	6107	5884	5928
Forth	Number of cataract surgeries	240,199	254,331	280,180	312,950	247,265	296,565
	Population (1000)	29,127	29,545	30,403	30,843	29,336	30,623
	CSR per one million	8247	8608	9216	10,147	8427	9681

^a^Average of 2012 and 2013

^b^Average of 2015 and 2016

**Fig. 1:**
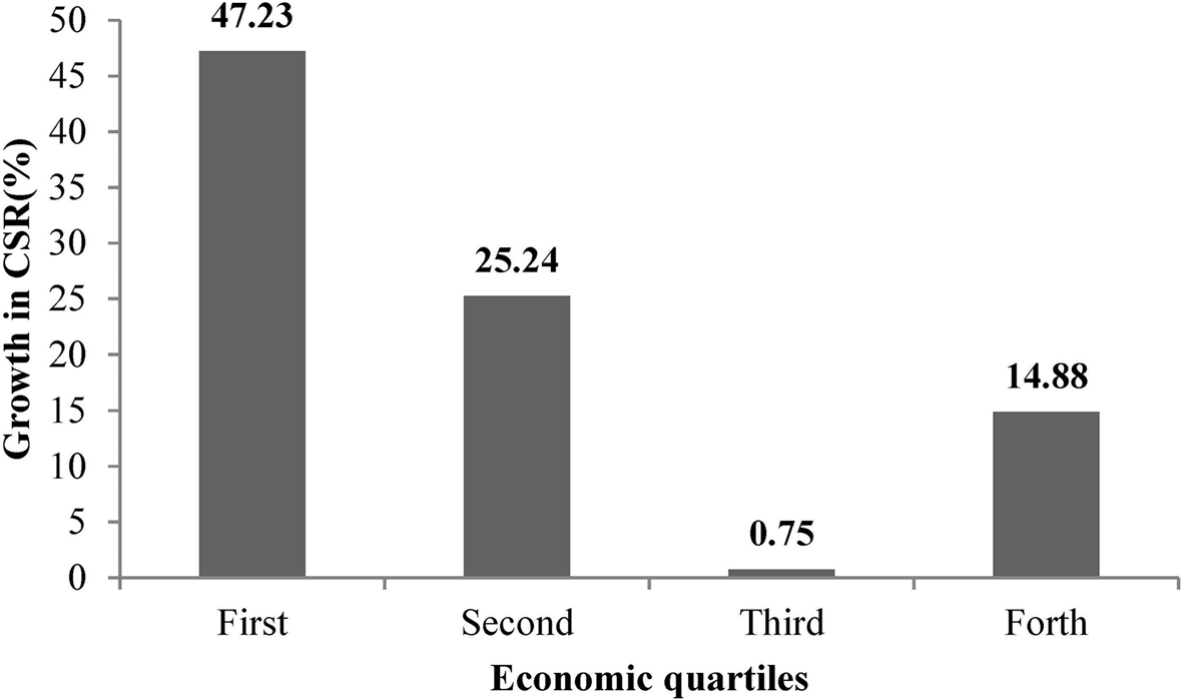
The growth in cataract surgical rate (CSR) according economic quartiles before (2012 and 2013 years) and after (2015 and 2016 years) Health Transformation Plan

Table 1 presents the number of cataract surgeries and CSR according to economic quartiles. According to the results, the number of cataract surgeries and CSR increased in all quartiles. In 2016, the highest CSR was in the 4^th^ and the lowest was in the 2^nd^ quartile. The number of cataract surgeries increased by 19.94%, 5.05%, 29.54%, and 49.33% in the 4^th^, 3^rd^, 2^nd^, and 1^st^ quartile, respectively. Figure 1 described the growth in CSR in the 1^st^ to 4^th^ quartiles. The largest growth in CSR occurred in the 1^st^ quartile followed by the 2^nd^, 4^th^, and 3^rd^ quartiles.

Table 2 presents the number of cataract surgeries before and after HTP according to the type of the cataract surgery center. The number of cataract surgeries increased from 221,814 surgeries before HTP to 321,767 surgeries after HTP in governmental centers (45.06% growth). The number of cataract surgeries increased by 1.79% in private centers, 9.9% in insurance centers, and 1.07% in charity centers after HTP.

**Table 2:** Number of cataract surgeries in Iran before and after Health Transformation Plan (HTP) according to the type of the cataract surgery center

Type of center	Before HTP		After HTP			
	2012	2013	2015	2016	Before HTP^a^	After HTP^b^
Governmental	213,220	230,407	292,680	350,854	221,814	321,767
Private	195,335	200,250	192,976	195,497	197,793	194,237
Insurance	43,415	46,887	39,623	41,720	45,151	40,672
Charity	10,134	9959	10,573	9735	10,047	10,154
Total	462,104	487,503	535,852	597,806	474,804	566,829

^a^Average of 2012 and 2013

^b^Average of 2015 and 2016

**Fig. 2:**
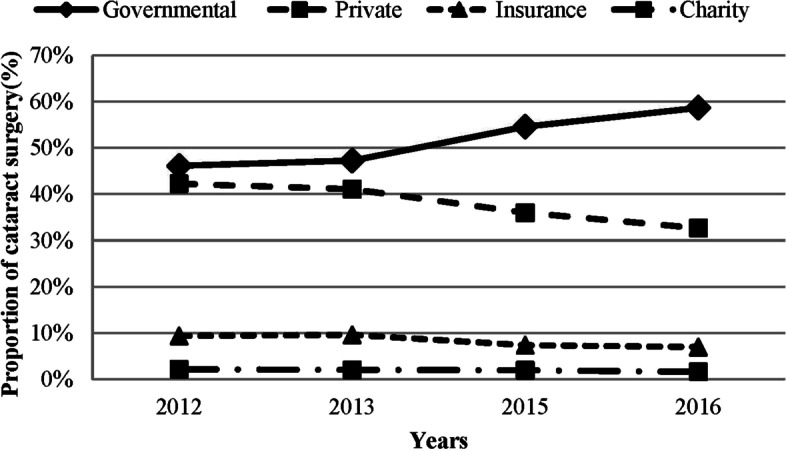
The proportion (%) of cataract surgeries according to the type of centers before (2012 and 2013 years) and after (2015 and 2016 years) Health Transformation Plan

Figure 2 describes the proportion of cataract surgeries according to the type of center. In 2012, 46.14%, 42.27%, 9.4%, and 2.19% of the cataract surgeries were performed in state, private, insurance, and charity centers, respectively. In 2016, the proportion of cataract surgery was 58.69% in governmental, 32.70% in private, 6.98% in insurance, and 1.63% in charity centers. In general, the mean proportion of cataract surgery before and after HTP was 46.70% and 56.66% in governmental centers, 41.67% and 36.34% in private centers, 9.51% and 7.19% in insurance centers, and 2.12% and 1.80% in charity centers. The proportion of cataract surgery increased by 21.32% in government centers and reduced by 17.56%, 24.45%, and 14.89% in private, insurance, and charity centers, respectively.

Table 3 presents the mean of uncorrected visual acuity before surgery (UCVA-BS) before and after HTP. There was no significant difference in the mean UCVA-BS between centers before HTP. However, after HTP, the worst UCVA-BS was seen in government centers and the best UCVA-BS was seen in private centers. Moreover, UCVA-BS decreased significantly only in government centers after HTP. Table 3 presents the results of UCVA-BS according to economic quartiles. According to the results, UCVA-BS decreased in the 1^st^ and 2^nd^ quartiles After HTP.

**Table 3:** The mean and standard deviation (SD) of uncorrected visual acuity before surgery (UCVA-BS) in LogMar before and after Health Transformation Plan (HTP) according to type of the cataract surgery center and economic quartiles

		Number of centers	Before HTP	After HTP	*p*-value
			Mean ± SD	Mean ± SD	
Cataract surgery center	Governmental	44	1.38 ± 0.52	1.21 ± 0.59	0.001
	Private	35	1.32 ± 0.44	1.31 ± 0.49	0.688
	Insurance	17	1.32 ± 0.57	1.33 ± 0.66	0.833
	Charity	3	1.39 ± 1.20	1.40 ± 0.92	0.960
Economic quartiles	First	9	1.41 ± 0.83	1.63 ± 1.13	0.227
	Second	22	1.36 ± 0.71	1.47 ± 0.80	0.058
	Third	30	1.34 ± 0.61	1.32 ± 0.77	0.590
	Forth	38	1.34 ± 0.48	1.32 ± 0.47	0.377

## Discussion

The CSR increased from 6074 cases per one million population in 2012 to 7134 cases per one million population in 2016, indicating a more than 15% growth in the CSR after HTP implementation. Although the overall CSR following HTP is high compared to several studies [12, 26], it is still low in comparison with countries like the USA [15], France [16], and Japan [26], which should receive prompt attention due to population ageing. An increase of about 10% in CSR has been reported from the US and France [3]. Although population ageing and increased demand for cataract surgery are effective in this regard, the CSR increased by about 25% in 2016 compared to 2012, which is not proportionate to the trend of population growth.

In 2012, the proportion of cataract surgery in private and governmental centers was almost similar (46.14 vs. 42.27) while it increased to 58.69% in governmental centers (22% increase) and reduced to 32.70% in private centers (18% reduction) in 2016.

A previous report from Iran showed that the proportion of cataract surgery was decreasing in governmental centers and increasing in private centers during 2006–2010 [27]. These findings clearly indicate that HTP implementation resulted in a marked increase in the proportion of cataract surgeries in governmental centers. It seems that in private centers, the supply and demand is a function of GDP, and the percentage of the rich reduced slightly due to GDP reduction.

An interesting finding was a reduction in the proportion of cataract surgeries in charity (15%) and insurance (25%) centers. Reduced GDP has a direct correlation with reduced insurance support [28]. Peleckienė et al. [28] reported a similar finding in European countries. In the present study, a 33% reduction of GDP was associated with a 25% decrease in the proportion of cataract surgeries in insurance centers. It seems that due to the higher franchise of HTP in terms of surgical costs compared to insurances or even charity centers, a large proportion of patients preferred to undergo cataract surgery in governmental centers. A CSR of 10,000 was reported from US in 2011 with a GDP of about 50,000$ [12], while Iran’s GDP was 5253$ and CSR was 7479 in 2016 with a high percentage of cataract surgeries being financially taken care of by the government. In other words, the results showed that the financial support of HTP for cataract surgery in government centers compensated the decreasing trend of GDP.

The highest growth in CSR was observed in the 1^st^ quartile followed by the 4^th^ quartile. The number of cataract surgeries was smaller in provinces in the 1^st^ quartile as poorer provinces due to the unavailability of eye care services (surgeon, IOL, surgical equipment) and poverty. However, the number of cataract surgeries increased markedly in these provinces after the HTP due to the government support for surgical costs. Hashemi et al. [29] also reported a lower CSR in poorer provinces. There are reports of economic inequity in cataract surgery and other eye care services in the literature [30]. It has even been shown that blindness and vision impairment are more prevalent in poorer populations with a high percentage of the difference between the poor and rich being due to the direct effect of the economic state [31, 32]. In other words, if blindness is considered an important outcome of leaving cataract untreated, a great part of the distribution of blindness is related to the economic state and inequity in the distribution of cataract surgical services between the 1^st^ and 4^th^ quartiles. After the 1^st^ quartile, the largest growth in the CSR occurred in the 2^nd^ quartile, which confirms the above.

Wang et al. [12] reported that surgical services were mostly offered by private centers and the patients pay for the services in high-income countries. Carvalho et al. [33] found that the highest concentration of ophthalmologists was in high-GDP areas in Brazil; therefore, part of CSR in private centers may be a function of GDP. Moreover, due to the high concentration of ophthalmologists in private centers, there is inequality in the distribution of cataract surgery, especially in economically weaker areas.

One of the HTP strategies was equity in the distribution of ophthalmologists, especially in the first 1^st^ economic quartile.

This strategy was implemented to support the retention of physicians in underserved areas and the family physician program. In this part, health houses and centers were equipped and physician coverage increased to 100% in these areas. Therefore, the CSR changes were greatest in centers in the 1^st^ and 4^th^ quartiles for two reasons, including the financial support of HTP for cataract surgery, which encouraged low-income people to undergo surgery, and increased proportion of ophthalmologists in centers in quartile one compared to before HTP implementation.

As mentioned earlier, UCVA-BS decreased slightly in charity enters compared to other enters before HTP, while the worst UCVA-BS after HTP was seen in government centers. Moreover, the mean UCVA-BS reduced significantly in government centers. It should be noted that some countries have certain visual acuity cut points for cataract surgery [34]. However, a worse UCVA-BS after HTP in patients presenting to government centers indicates that subjects with cataract related vision impairment that could not afford surgery due to financial constraints, were on long waiting lists, and thus had lost their vision decided to undergo surgery after HTP. Other reasons may also explain this finding. First, people also sought treatment before HTP but the system could not respond to the high demand for treatment, and therefore people were on long waiting lists and lost their vision. Second, since access to treatment and surgery was facilitated after HTP, there was an increase in the demand for cataract surgery, resulting in vision loss in people on long waiting lists.

The results showed that UCVA-BS became worse in patients in the 1^st^ and 2^nd^ quartiles that presented for surgery after HTP.

In other words, the patients in these quartiles, as poor quartiles, waited long for surgery due to financial problems and lost their vision before HTP; however, a high percentage of them decided to undergo surgery after HTP resulting in a marked reduction in UCVA-BS after HTP. Since the data were analyzed two years after implementing the HTP, this finding indicates the immediate effect of HTP on vision impairment. Population-based studies in longer periods are required to evaluate the long-term effect of HTP on vision impairment in different populations to investigate the prevalence of vision impairment after HTP.

UCVA-BS improved slightly in private centers and high-income quartiles. It seems that the high percentage of patients presenting to governmental centers for surgery and reduced demand for surgery in private centers after HTP led to overtreatment in these patients; in other words, patients with a better visual acuity were received surgery in these centers.

## Data Availability

The datasets used and/or analysed during the current study available from the corresponding author on reasonable request.
